# Paraphrenia: a lost concept

**DOI:** 10.1192/j.eurpsy.2022.2032

**Published:** 2022-09-01

**Authors:** S. Vicent Forés, S. Sainz De La Cuesta Alonso, F. Calera Cortés, T. Gutierrez Higueras

**Affiliations:** 1 Hospital Reina Sofía Córdoba, Psychiatry, Córdoba, Spain; 2 Hospital Reina Sofía Córdoba, Psychiatry, Cordoba, Spain

**Keywords:** Paraphrenia, Chronic delusion, Classic termionolgy, Expansive discourse

## Abstract

**Introduction:**

Paraphrenia consists on a syndrome of insidious development with a chronic delirium of great phenomenological richness, predominating productive or delusional-hallucinatory forms and with time it evolves to pure fabulation. Delusions appear in 100% of cases predominating persecution, reference and false identifications. It is a classic term that disappeared with DSM-III, but is still useful for the description of certain clinical cases.

**Objectives:**

Presentation of a case that clearly defines the classic term paraphrenia, which is now a days lost in new classifications.

**Methods:**

We carried out a literature review of the term paraphrenia and presented a real case of a patient interned in our psychiatric ward.

**Results:**

A 55-year-old woman, was without treatment or attendance to her psychiatrist for years, admitted to the hospital due to public disturbance. Even the lack of treatment did not repercuss greatly emotionally or behaviorally. During our interviews, she showed an expansive discourse rich in delirious content, as well as thought transmission and reading, auditive hallucinations and corporal influence. As we can see, this case exposes what would have classical been classified as a case of paraphrenia, nowadays we cannot find a better term to name this group of symptoms with the current classifications.

**Conclusions:**

We can conclude that paraphrenia is halfway between schizophrenic disorganization and paranoic structuring. The personal deterioration is significantly lower than in schizophrenia and the expression of delirium differs from paranoia. Even though actual classifications provide simplicity and pragmatism, we risk losing the semiological and phenomenological richness of classic terminology.

**Disclosure:**

No significant relationships.

